# Supplementary material for: Estimation of immune cell content in tumour tissue using single-cell RNA-seq data
Source: Nat Commun. 2017 Dec 11;8:2032. doi: 10.1038/s41467-017-02289-3 (PMC5725570; doi:10.1038/s41467-017-02289-3)
Supplement: Supplementary file 1 — Supplementary Information [file 41467_2017_2289_MOESM1_ESM.pdf]

	Classification (single-cell)			Deconvolution (bulk)			FACS (single-cell)		
**Cell types/ Sample ID**	**Ascites 7873 M**	**Ascites 7882 M**	**Ascites 7892 M**	**Ascites 7873 M**	**Ascites 7882 M**	**Ascites 7892 M**	**Ascites 7873 M**	**Ascites 7882 M**	**Ascites 7892 M**
CD4+ T cells	3%	15%	12%	2%	6%	8%	8%	16%	12%
CD8+ T cells	0%	2%	1%	3%	2%	4%	2%	4%	3%
regulatory T cells	0%	0%	0%	1%	1%	0%	0%	1%	0%
B cells	1%	0%	1%	0%	0%	2%	0%	0%	0%
Macrophages/ Monocytes	85%	64%	65%	77%	55%	47%	68%	33%	missing
Dendritic cells	3%	5%	5%	0%	0%	0%	3%	3%	2%
Natural killer cells	1%	5%	5%	0%	0%	0%	2%	3%	0%

Supplementary Table 1: Estimates of cellular composition by three different methods. Three ovarian cancer ascites samples were profiled by single-cell and bulk RNA sequencing as well as FACS. Estimates of the cellular composition are derived by: 1) Classification based on single-cell RNA sequencing data; 2) Computational deconvolution on the bulk RNA sequencing data using the single-cell RNA sequencing derived RGEP3; 3) Quantification by FACS. For sample 7892, macrophages/monocytes quantification could not be determined by FACS.

	Classification (single-cell)			Deconvolution (bulk)		
**Cell types/ Sample ID**	**Ascites 7873M**	**Ascites 7882M**	**Ascites 7892M**	**Ascites 7873M**	**Ascites 7882M**	**Ascites 7892M**
CD4+ T cells	-5%	-1%	0%	-5%	-9%	-4%
CD8+ T cells	-2%	-2%	-2%	1%	-2%	2%
regulatory T cells	0%	0%	0%	1%	0%	0%
B cells	0%	0%	1%	0%	0%	2%
Macrophages/ Monocytes	17%	31%	missing	9%	22%	missing
Dendritic cells	0%	2%	3%	-3%	-3%	-2%
Natural killer cells	-1%	2%	5%	-2%	-3%	0%

Supplementary Table 2: Deviation of estimates of cellular composition relative to FACS method.

Estimates of the cellular composition for each method are shown in Suppl. Tab. 2. For sample 7892, macrophages/monocytes quantification could not be determined by FACS.

Cell type	Required marker gene (AND)	Optional marker gene (OR)	Marker gene that must be absent (NOT)
T cells	CD3D, CD3E, CD3G, CD27, CD28		
CD4+ T cells	CD4		FOXP3, IL2RA, CTLA4
CD8+ T cells	CD8B	CD8A	CD4
Regulatory T cells	CD4, FOXP3, IL2RA, CTLA4		
B cell	CD19, MS4A1, CD79A, CD79B, BLNK		
Macrophages/ Monocyte	CD14, CD68, CD163, CSF1R, FCGR3A		
Dendritic cell	IL3RA, CLEC4C, NRP1		
Natural killer cells	FCGR3A, FCGR3B, NCAM1, KLRB1, KLRB1, KLRC1, KLRD1, KLRF1, KLRK1		
Endothelial cell	VWF, CDH5, SELE		
Cancer associated fibroblasts	FAP, THY1, COL1A1, COL3A1		
Ovarian carcinoma cells	WFDC2, EPCAM, MCAM		
Melanoma cell	PMEL, MLANA, TYR, MITF		

Supplementary Table 3: Marker genes used to characterise unknown cell types from single-cell RNA-seq gene expression profiles.

The normalized gene expression of each marker gene is used for one of three logic gates to determine the overall expression for each cell type.

Supplementary Note 1: Problem definition for the CVX package for MATLAB

```
cvx_begin quiet
    cvx_solver sdpt3;
    variable w(size(B, 2)) nonnegative;
    minimize( norm((B*w - m), 2) + lambda*norm(f, 2))
    subject to
        w <= 1;
        sum(w) <= 1;
cvx_end
```

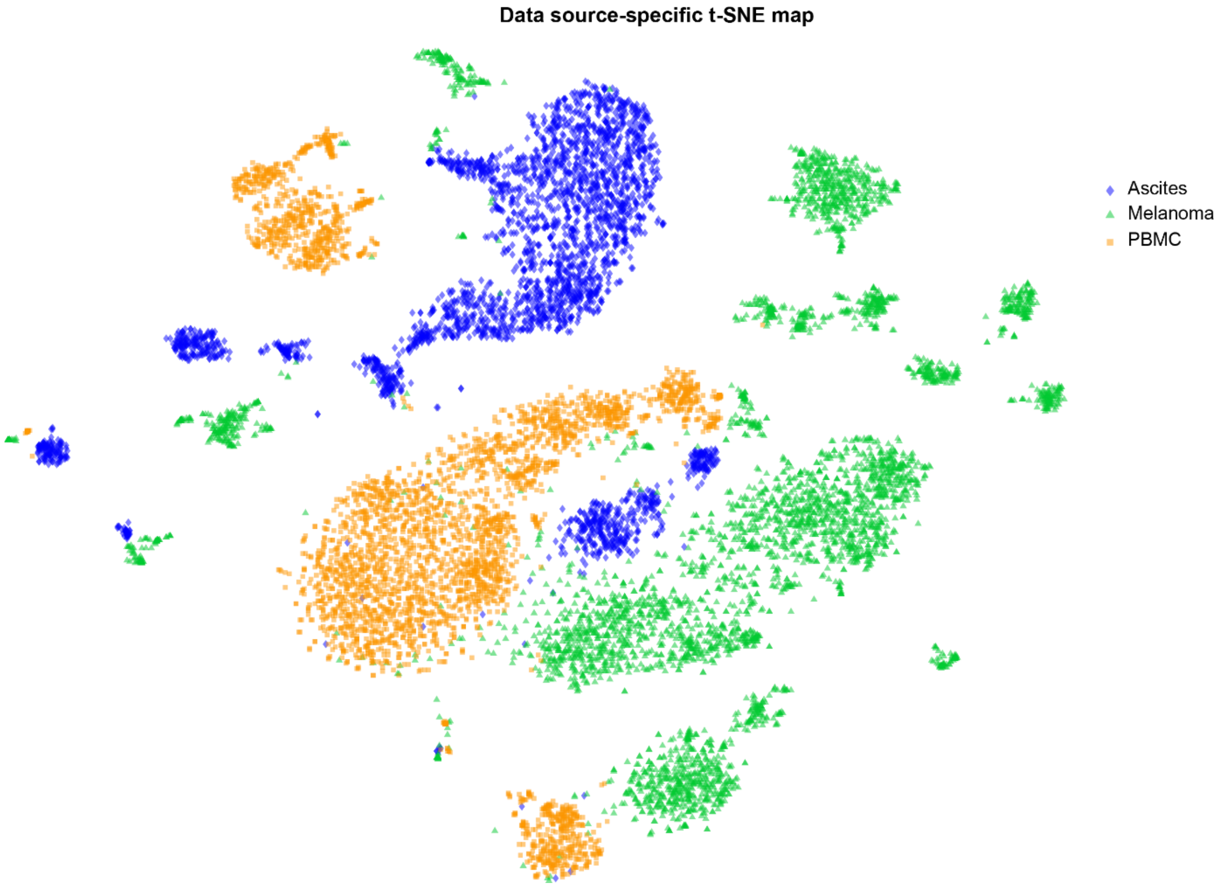

Supplementary Figure 1: t-SNE map with data source-specific colour-coding.
Single cells (symbols) were arranged in two dimensions based on similarity of their gene expression profiles by the dimensionality reduction technique t-SNE. Colours indicate the source location of each single cell (triangles for melanoma, squares for PBMCs, and diamonds for ascites).

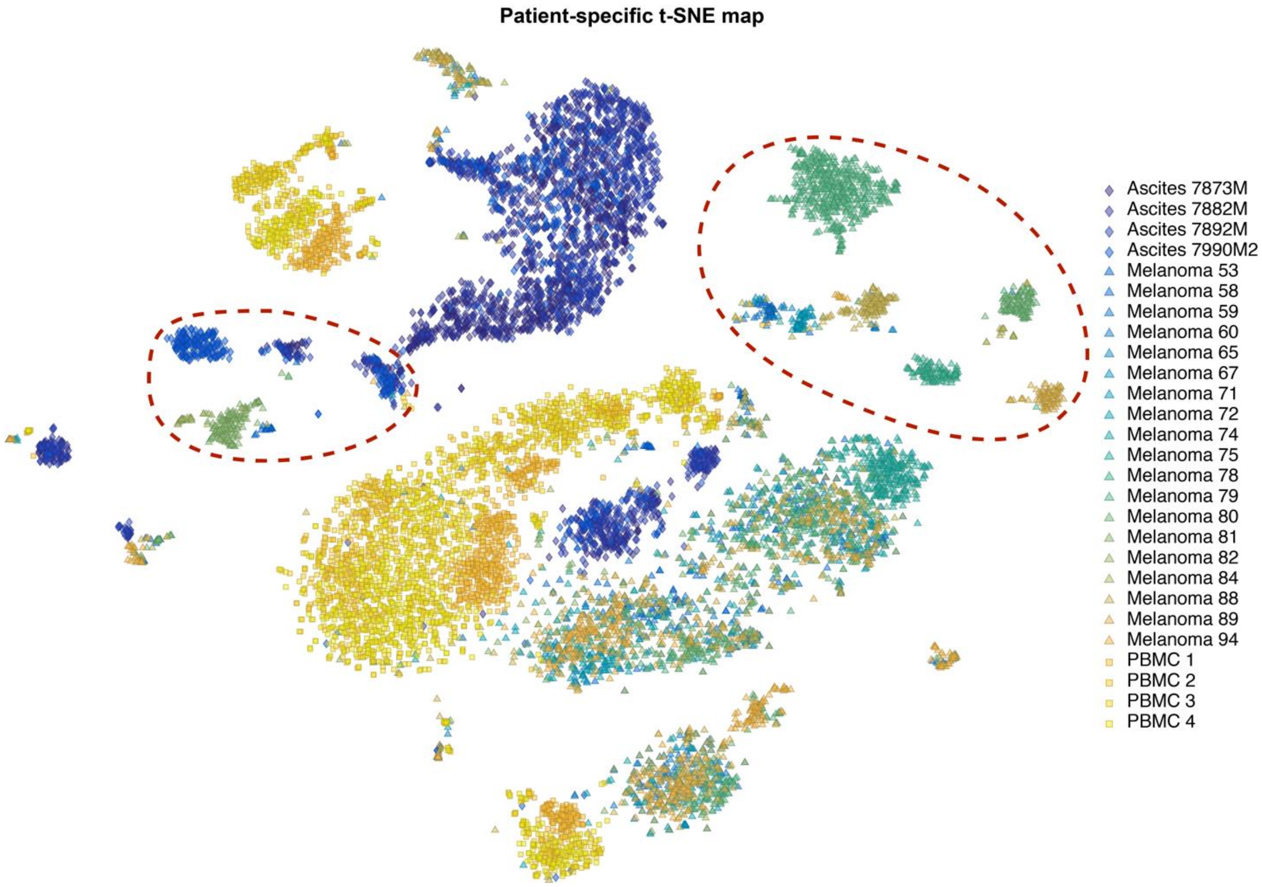

Supplementary Figure 2: t-SNE map with patient-specific colour-coding.
Single cells (symbols) were arranged in two dimensions based on similarity of their gene expression profiles by the dimensionality reduction technique t-SNE. Colours indicate the patient sample and symbols show the source location (triangles for melanoma, squares for PBMCs, and diamonds for ascites). Red dashed ellipses indicate clusters of malignant tumour cells.

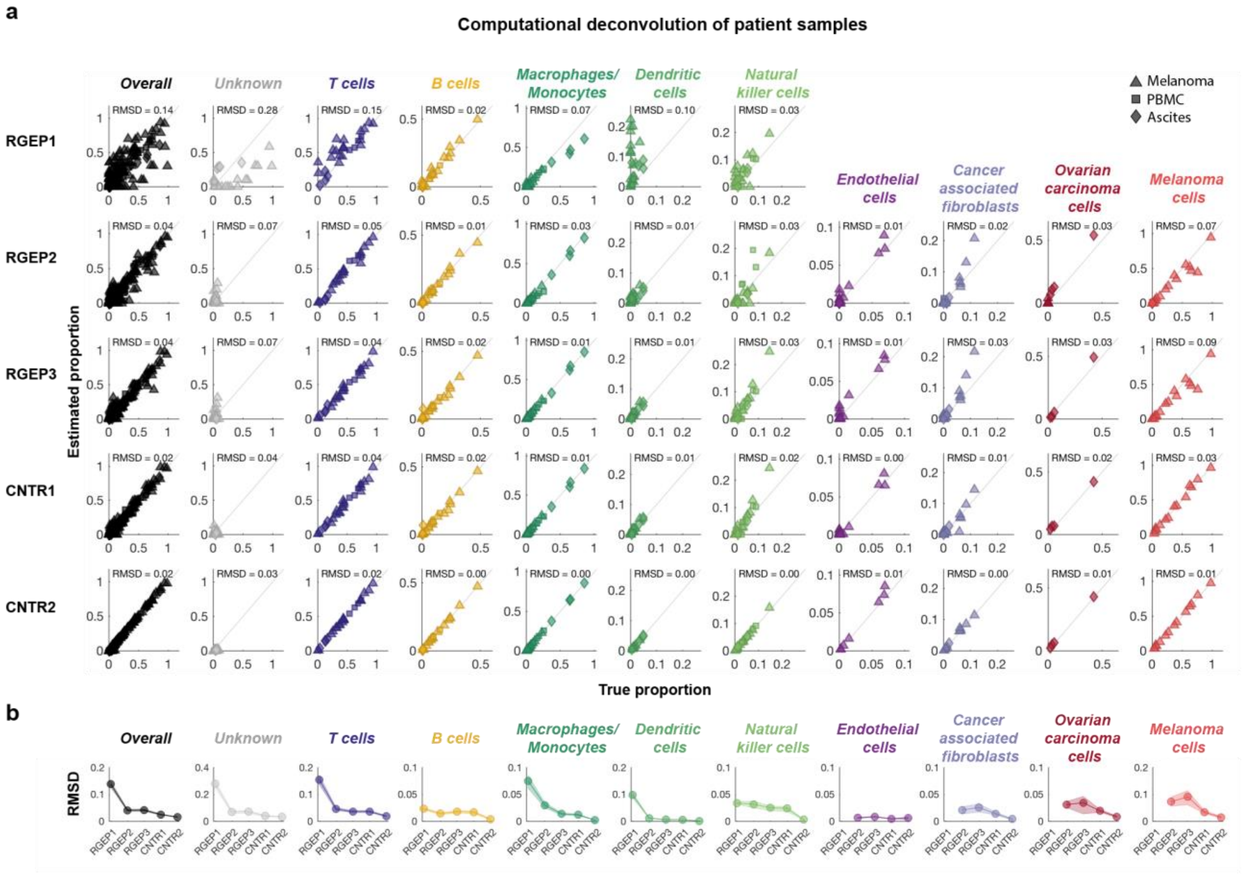

Supplementary Figure 3: Detailed deconvolution results based on root-mean-square deviation (RMSD) as measure of estimation quality. (a) Scatter plot of true and estimated cell proportions for all 27 patient samples. Each dot represents one patient sample. Values close to the diagonal correspond to high deconvolution accuracy. Columns depict cell types; rows describe the five different configurations (REGP1-3 and CNTR1-2). In configuration REGP1, estimates for tumour-associated cell types are not available. **(b)** RMSD for all five configurations. Dots denote the median RMSD; the shading represents the uncertainty based on bootstrapping (upper and lower quartile). (Please note the different scaling of the figure axes.)

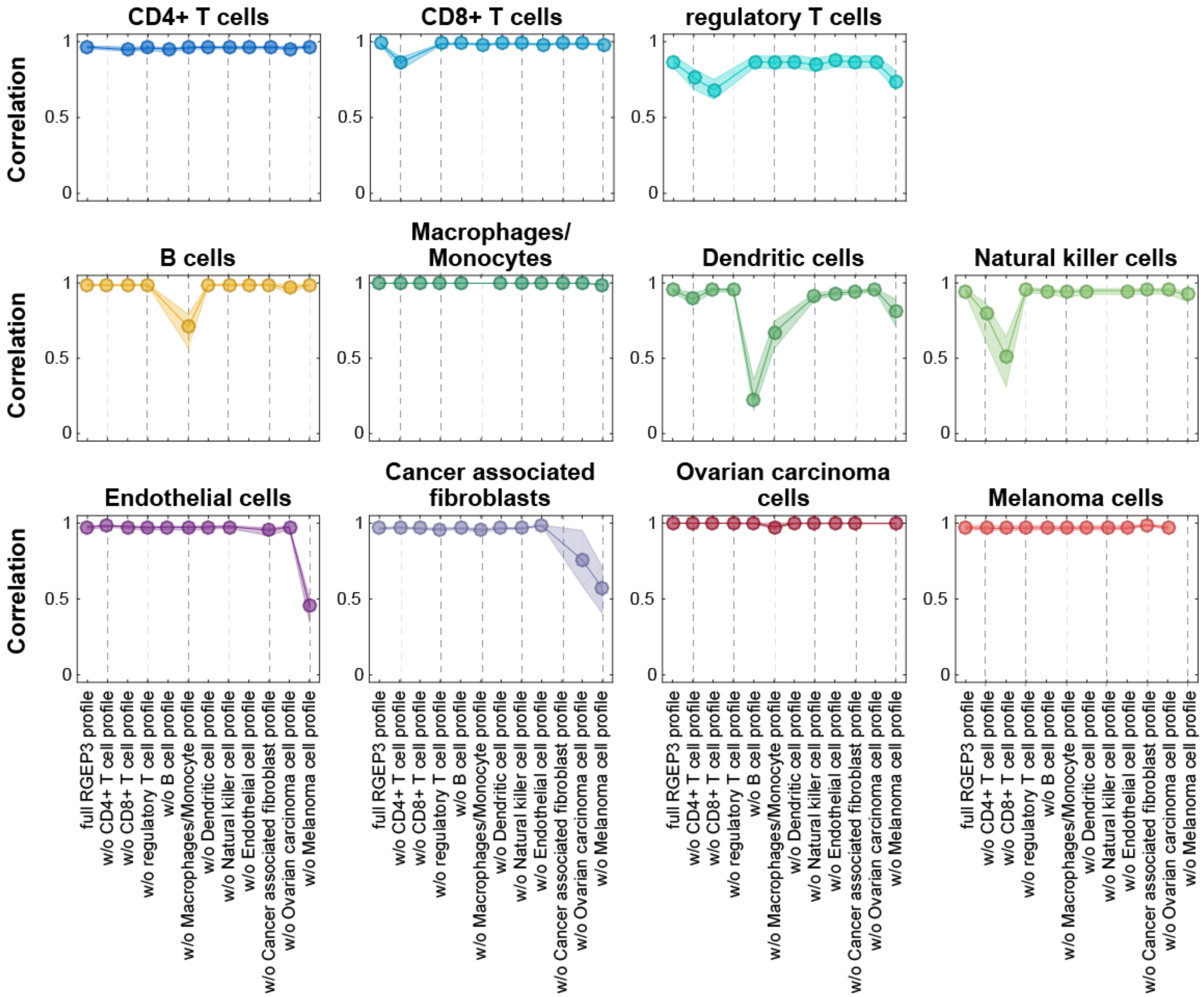

Supplementary Figure 4: Impact of missing cell types on estimation accuracy.
Dots denote the median of the correlation coefficient as measure of estimation accuracy; the shading represents the uncertainty based on bootstrapping (lower and upper quartile). For each cell type, the deconvolution accuracy for the full RGEP3 profile (left) is compared to a reduced RGEP3 leaving out each cell type at a time (to the right). For cases where certain profiles were removed from RGEP3 the corresponding estimation of this cell type is missing (e.g., the B cell estimation is missing for the case “w/o B cell profile”).

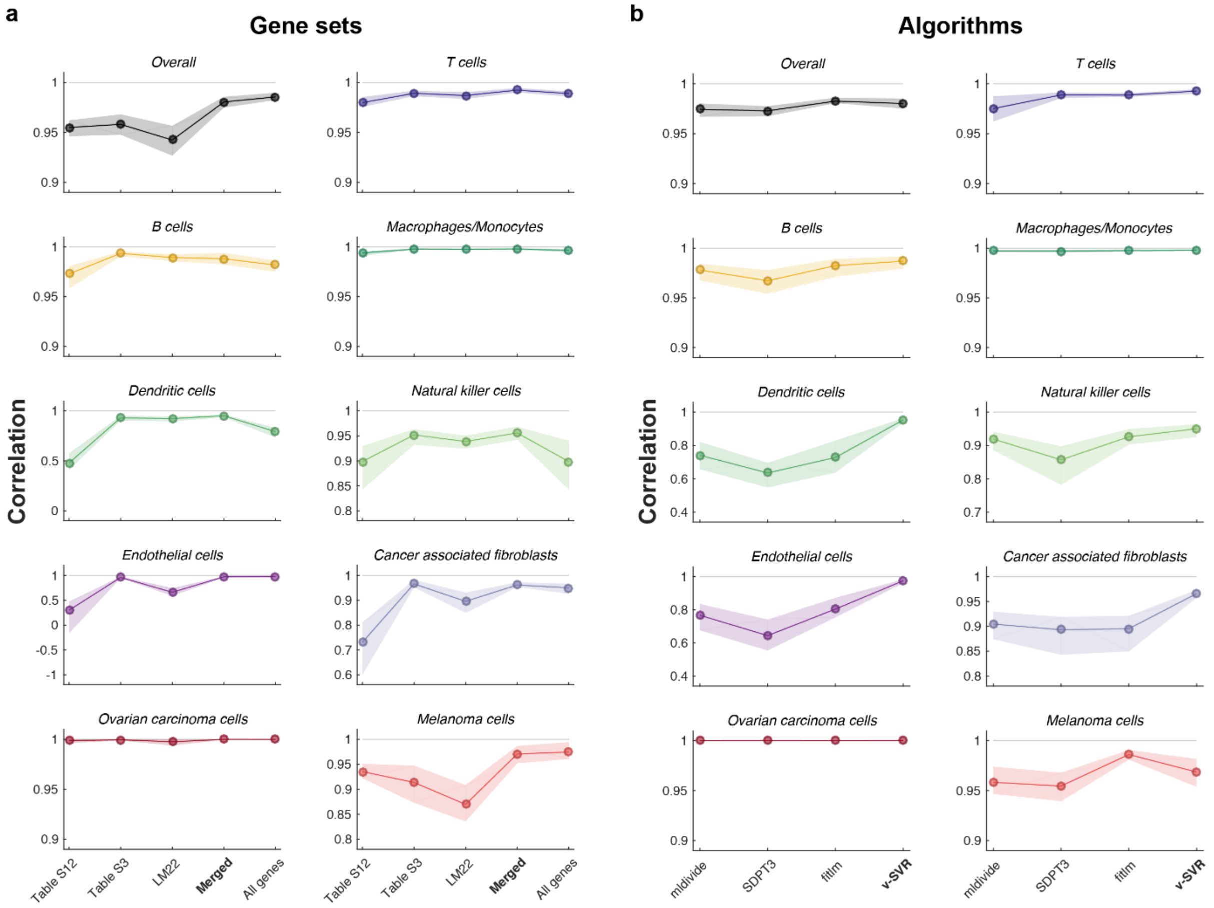

Supplementary Figure 5: Estimation accuracy is dependent on different signature gene sets and deconvolution algorithms.

Dots denote the median of the correlation coefficient as measure of estimation accuracy; the shading represents the uncertainty based on bootstrapping (lower and upper quartiles). Individual scatter plots are shown in Suppl. Fig. 8-14. **(a)** Comparison of five different signature gene sets used for generating the reference gene expression profiles. Table S12: a published set of 244 marker genes differentially expressed in regulatory T cell subpopulations based on the scRNA-seq melanoma data[11]; Table S3: a published set of 385 marker genes based on the scRNA-seq melanoma data[11]; LM22: gene set which comprises 547 signature genes that were found to maximally differentiate various cell types[8]; Merged: a list of 1076 unique genes combined from the LM22, Table S12 and Table S3 gene lists as well as the 45 marker genes used for classification; All genes: a list of 17,933 genes that have a non-zero expression in at least one sample. **(b)** Comparison of four different algorithms used in the deconvolution approach. mldivide: exact solution using an algorithm for matrix inversion in MATLAB (The MathWorks, Inc.); SDPT3: a semidefinite-quadratic-linear programming algorithm from the CVX package[23]; fitlm: fitting a linear model (y = a*x+b) to the data based on least-squares in MATLAB; ν-SVR: a support vector regression algorithm used in the CIBERSORT method.

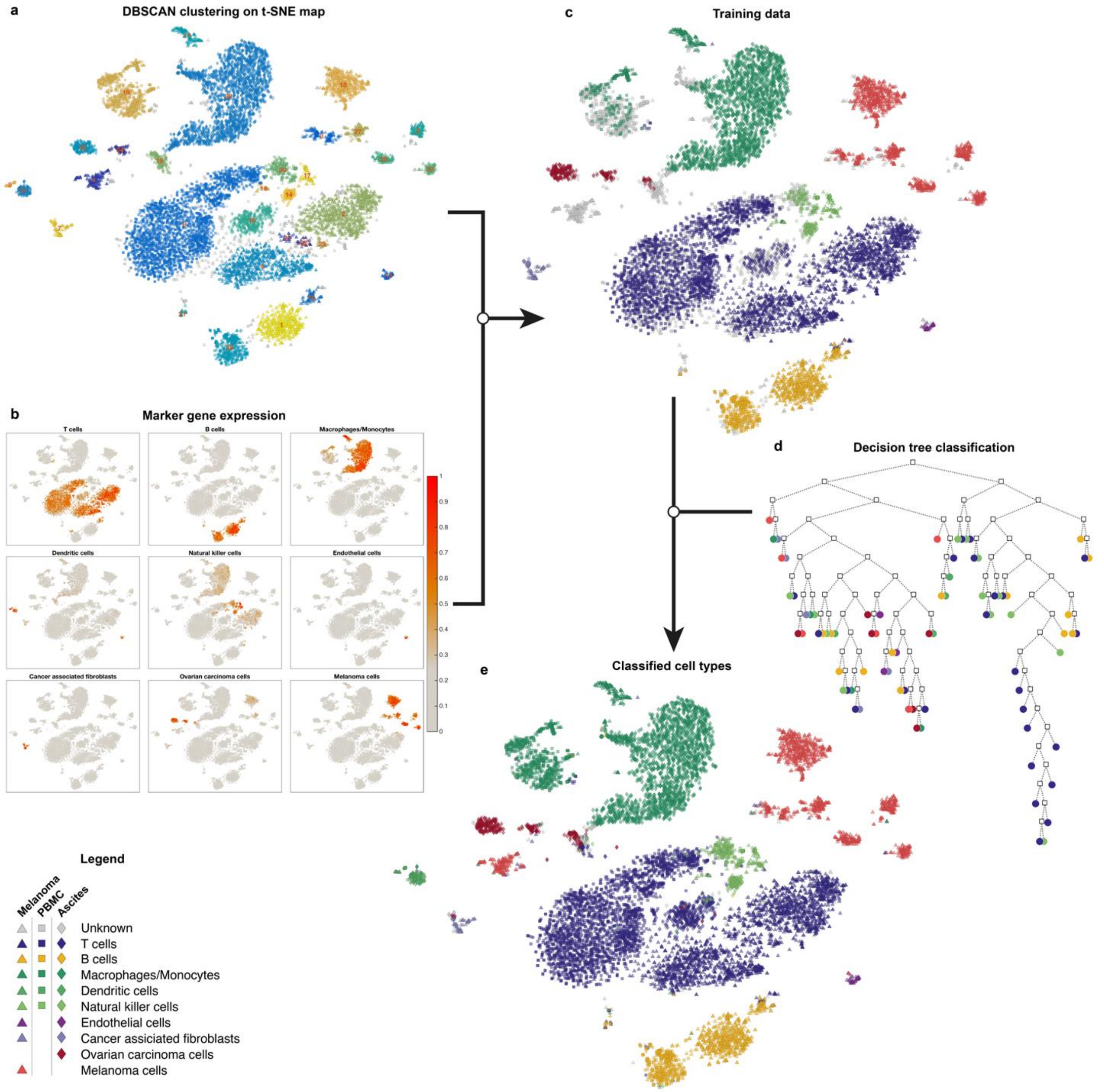

Supplementary Figure 6: Classification of cell types from scRNA-seq expression profiles using decision trees.

(a) DBSCAN clustering is performed on the t-SNE map to identify distinct cell clusters with high similarity. **(b)** The expression of 45 marker genes is evaluated based on three logical gates (AND, OR, NOT) and shown on top of the t-SNE mapping. **(c)** Predominant cell types within each cluster are identified based on the marker gene expression and used as a training set for unsupervised classification. **(d)** A decision tree classifier is trained and utilized to predict the cell types of all individual cells. **(e)** The resulting map indicates all nine major cell types by colour and by indication (melanoma, ascites) or location (PBMCs) by symbols.

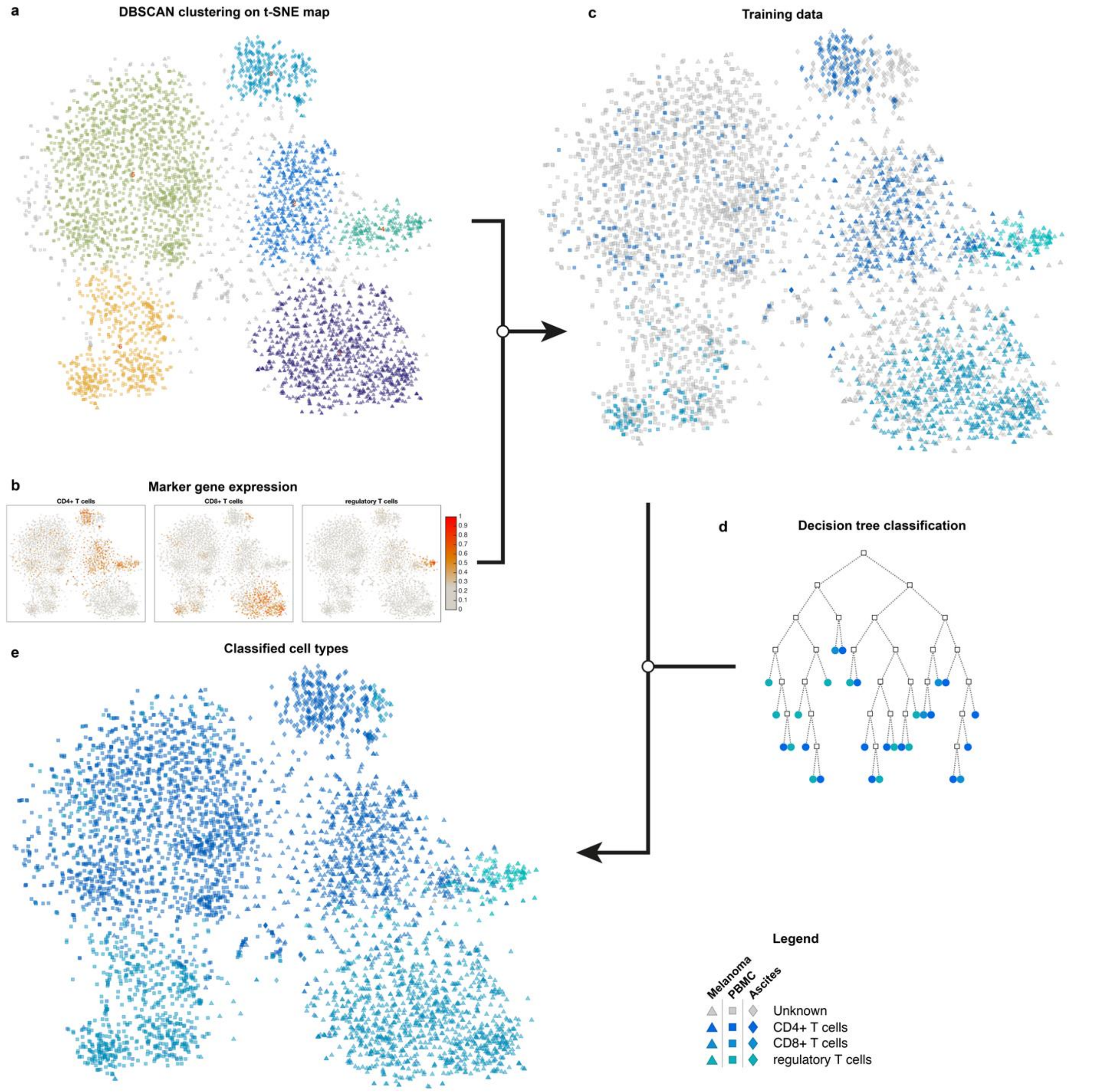

Supplementary Figure 7: Classification of T cell subtypes from scRNA-seq expression profiles.
(a) DBSCAN clustering is performed on the t-SNE map to identify distinct cell clusters with high similarity. **(b)** The expression of six T cell marker genes is evaluated based on three logical gates (AND, OR, NOT) and shown on top of the t-SNE mapping. **(c)** Predominant sub types within each cluster are identified based on the marker gene expression and used as a training set for unsupervised classification. **(d)** A decision tree classifier is trained and utilized to predict the subtype of all individual cells. **(e)** The resulting map indicates three subtypes of T cells by colours and the data source (melanoma, PBMC, ascites) by symbols.

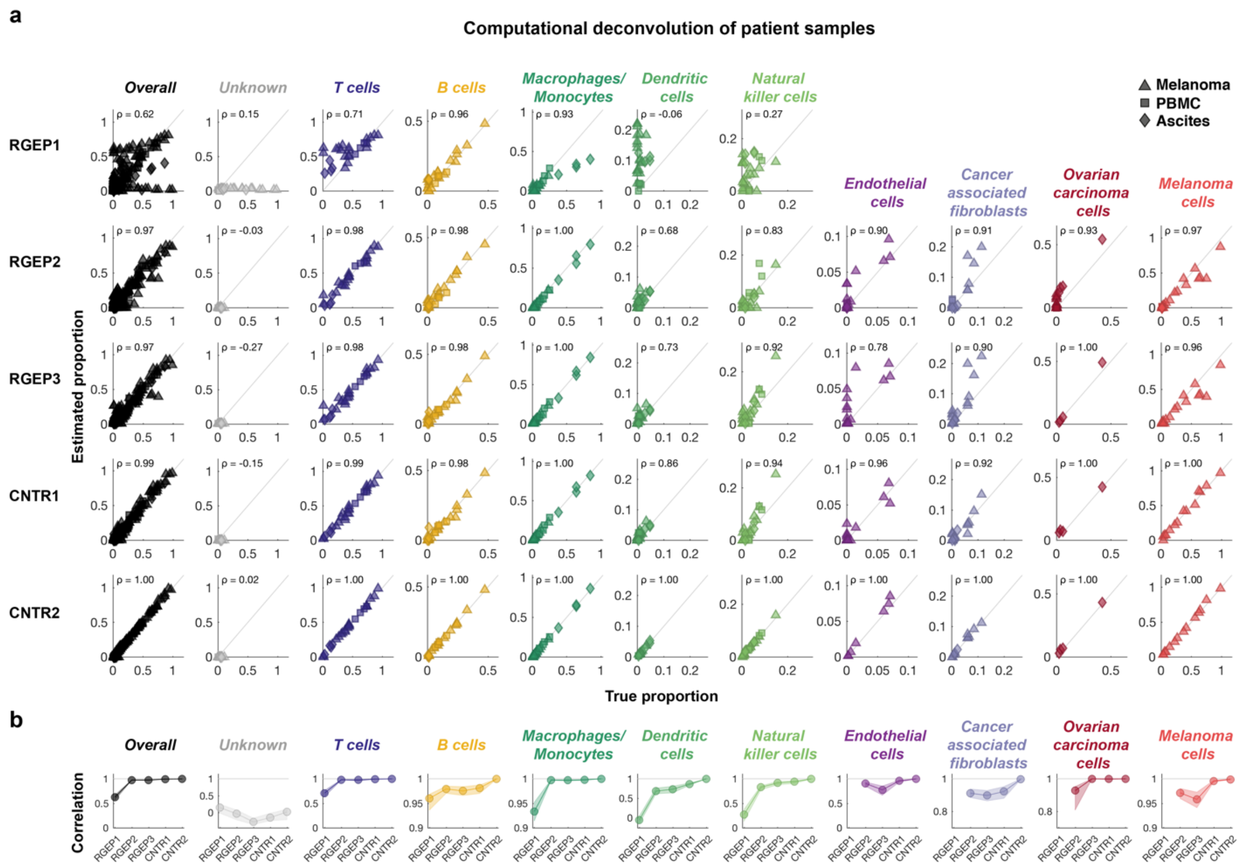

Supplementary Figure 8: Detailed deconvolution results based on the "mldivide" algorithm and the "Merged" gene set.

(a) Scatter plot of true and estimated cell proportions for all 27 patient samples. Each dot represents one patient sample. Values close to the diagonal correspond to high deconvolution accuracy. Columns depict cell types; rows describe the five different configurations (REGP1-3 and CNTR1-2). ρ denotes the Pearson's correlation coefficient. In configuration REGP1, estimates for tumour associated cell types are not available. **(b)** Pearson's correlation coefficient between estimated and true cell fraction for all five configurations. Dots denote the mean of the correlation coefficient; the shading represents the uncertainty based on bootstrapping. (Please note the different scaling of the figure axes.)

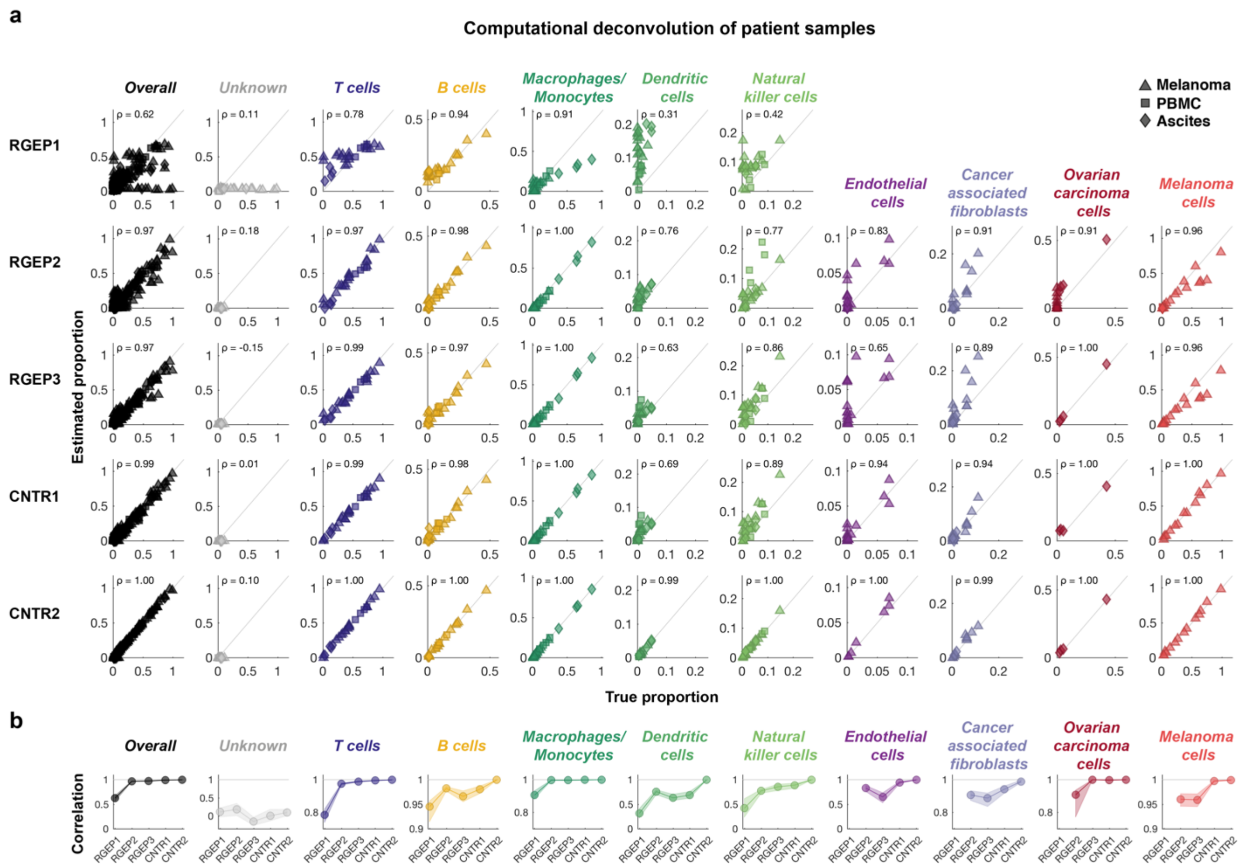

Supplementary Figure 9: Detailed deconvolution results based on the "SDPT3" algorithm and the "Merged" gene set.

Description as for Supplementary Figure 2.

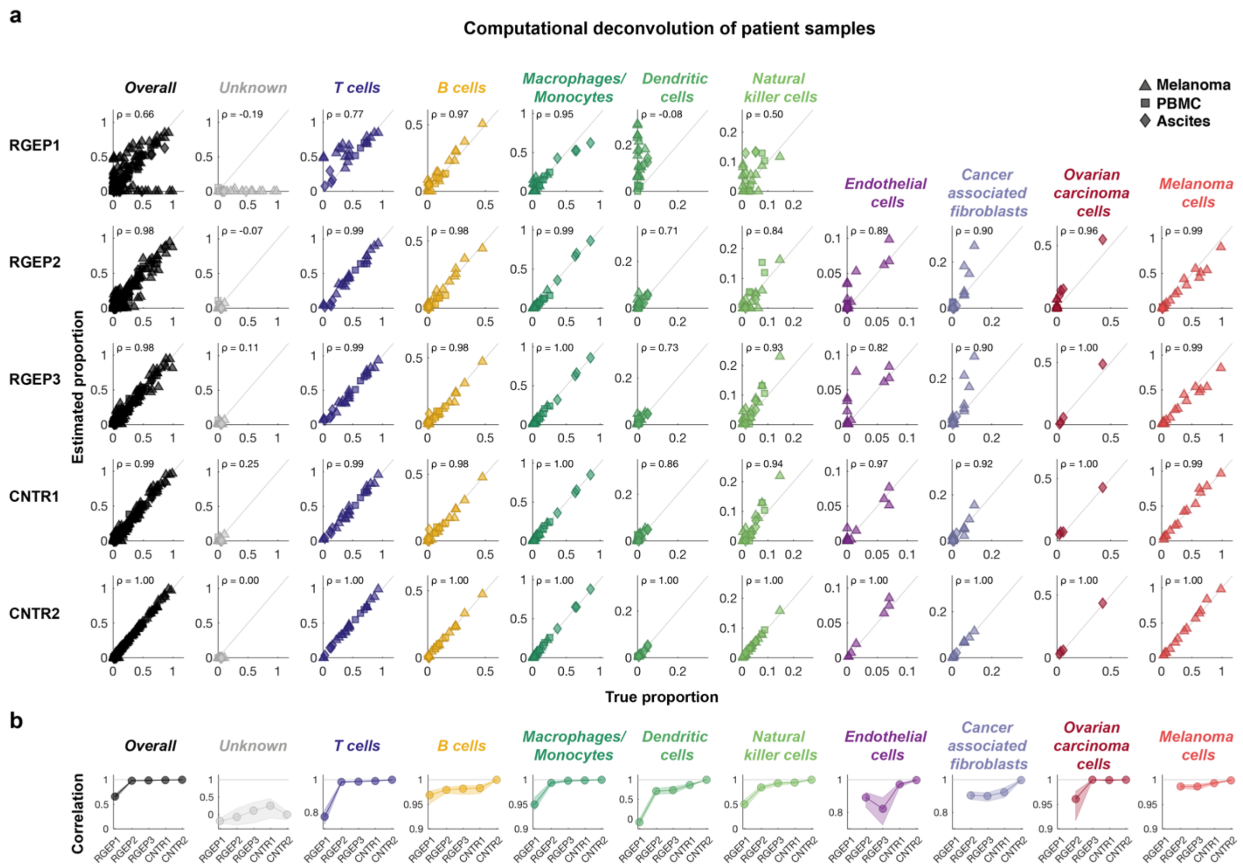

Supplementary Figure 10: Detailed deconvolution results based on the "fitlm" algorithm and the "Merged" gene set.
Description as for Supplementary Figure 2.

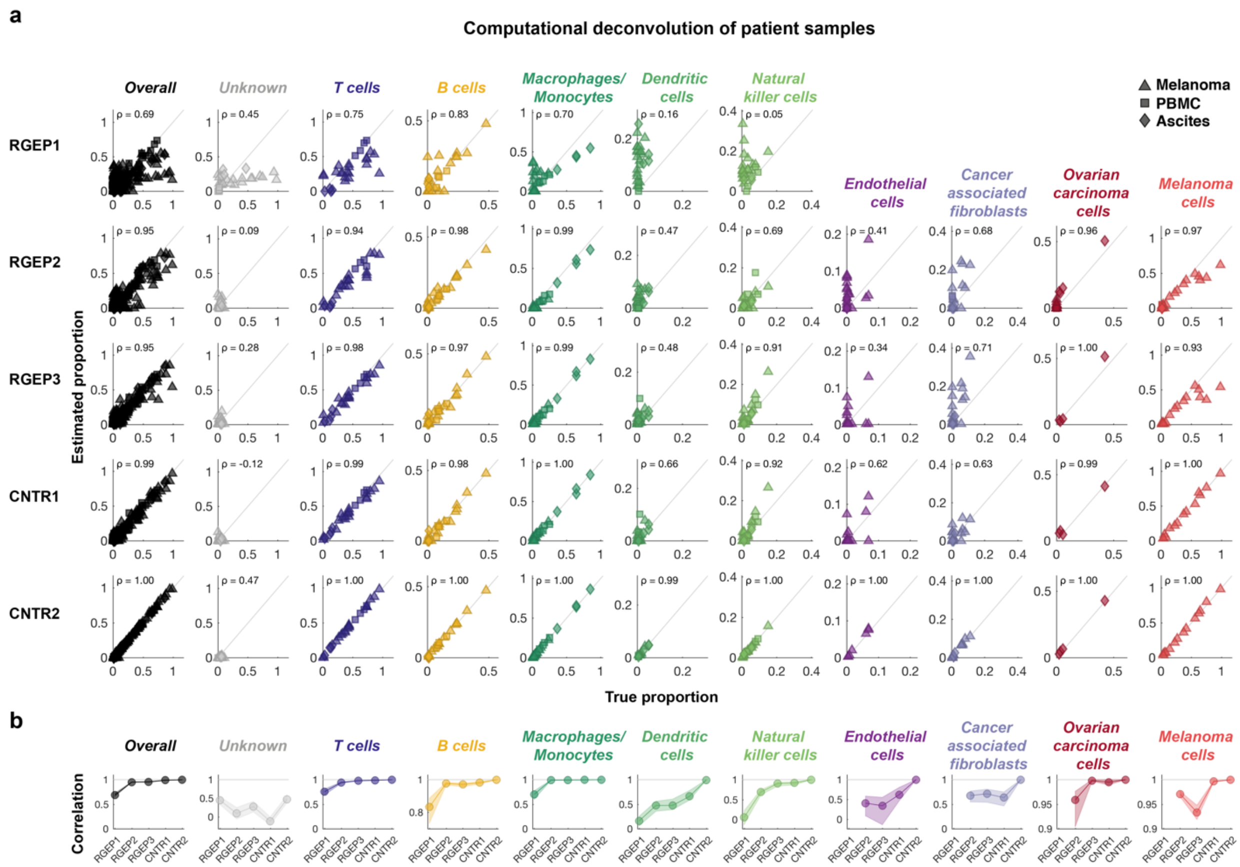

Supplementary Figure 11: Detailed deconvolution results based on the “Table S12” gene set and the “ν-SVR” algorithm.

Description as for Supplementary Figure 2.

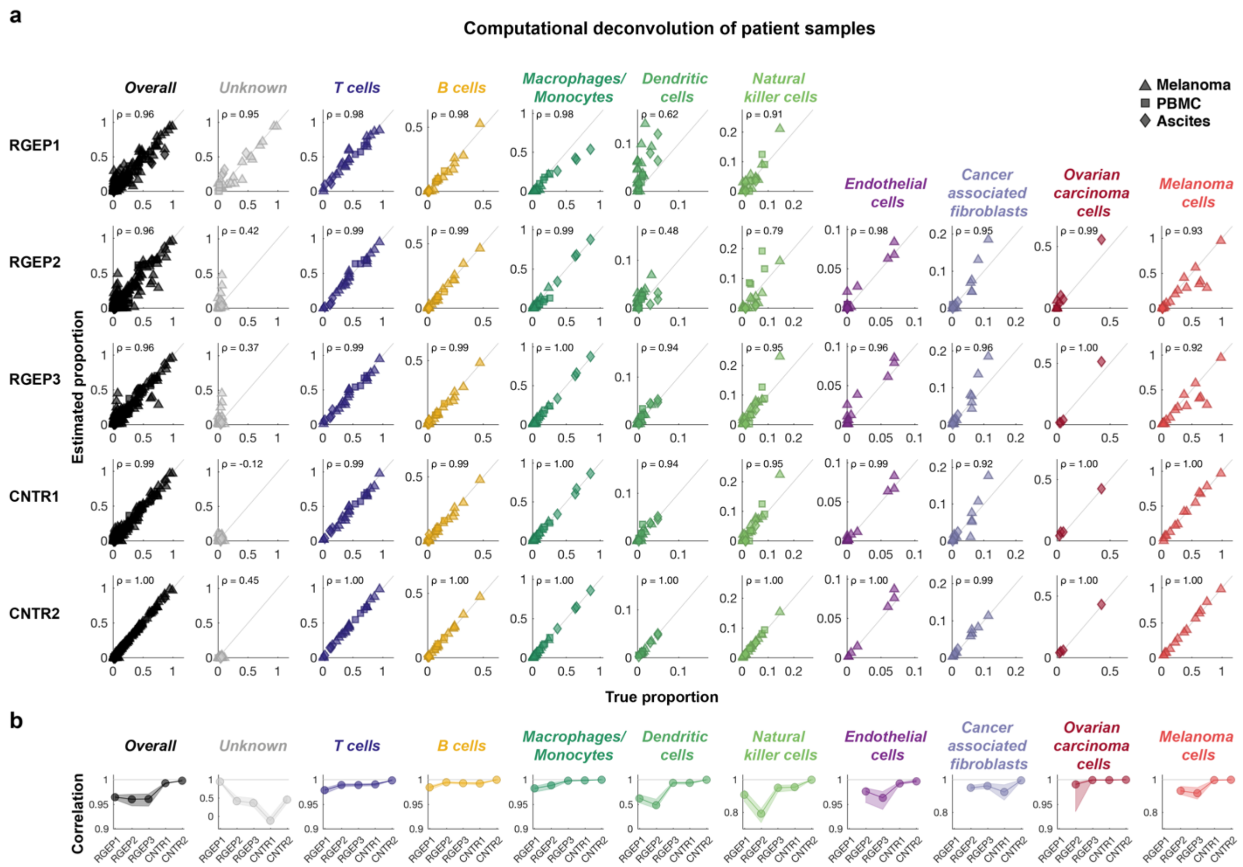

Supplementary Figure 12: Detailed deconvolution results based on the “Table S3” gene set and the “ν-SVR” algorithm.
Description as for Supplementary Figure 2.

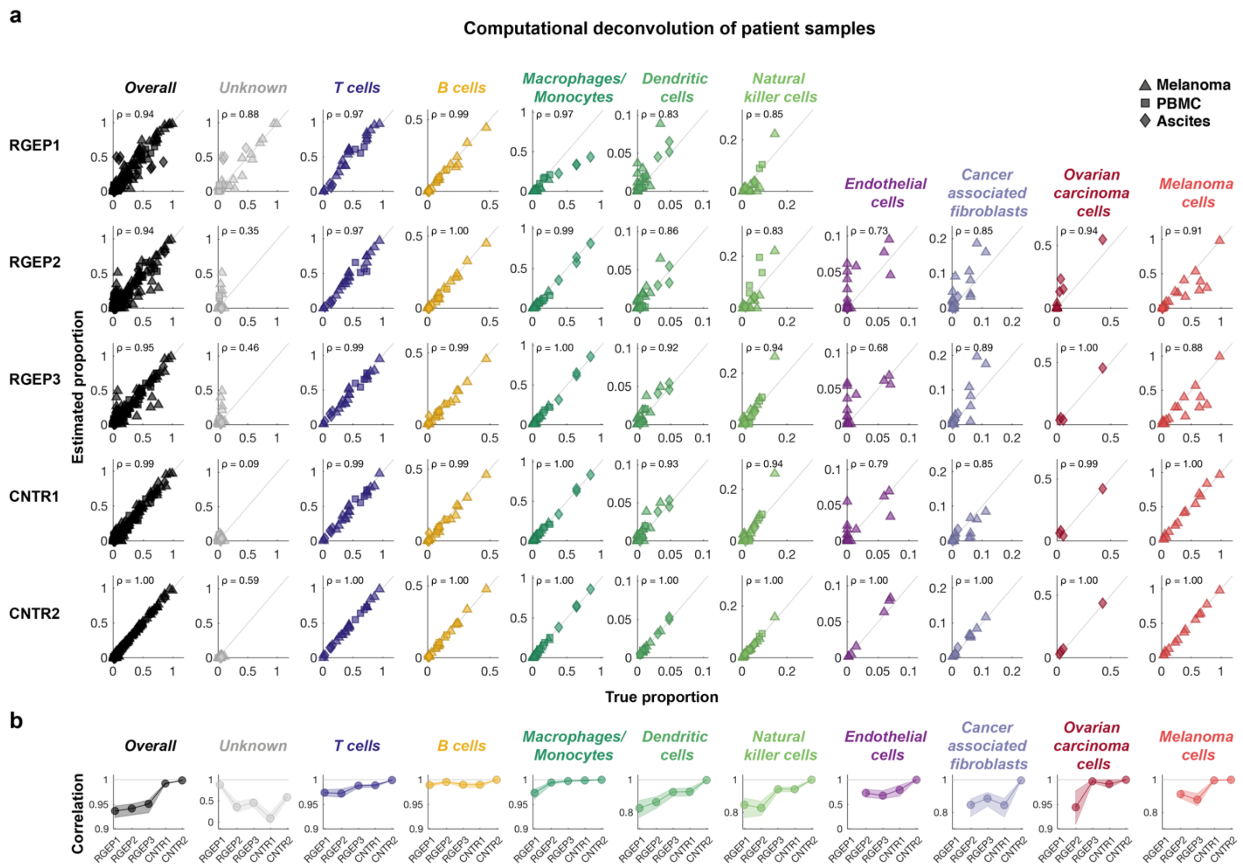

Supplementary Figure 13: Detailed deconvolution results based on the "LM22" gene set and the "ν-SVR" algorithm.

Description as for Supplementary Figure 2.

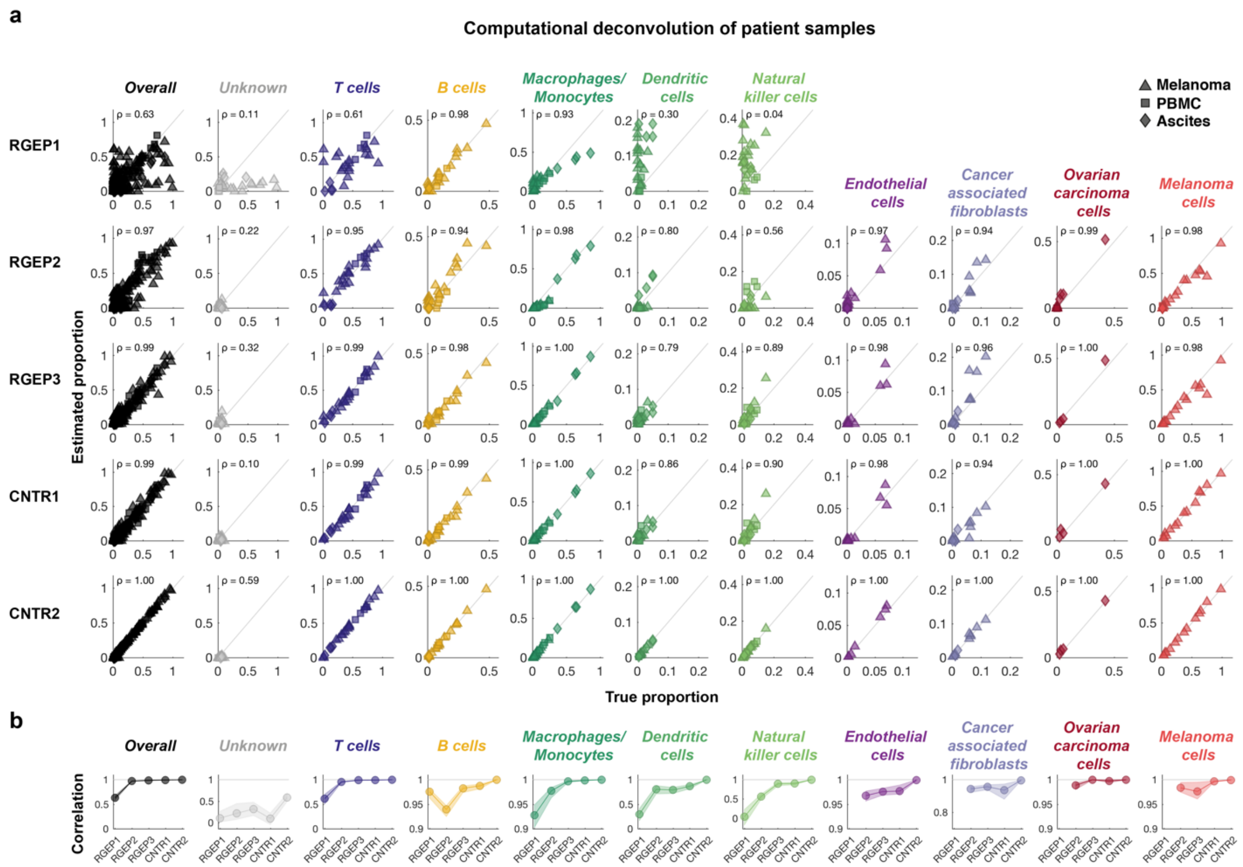

Supplementary Figure 14: Detailed deconvolution results based on the "All genes" gene set and the "ν-SVR" algorithm.

Description as for Supplementary Figure 2.
